# Affective Images of Climate Change

**DOI:** 10.3389/fpsyg.2019.00960

**Published:** 2019-05-15

**Authors:** Betsy Lehman, Jessica Thompson, Shawn Davis, Joshua M. Carlson

**Affiliations:** ^1^ Department of Psychological Science, Northern Michigan University, Marquette, MI, United States; ^2^ Department of Communication and Performance Studies, Northern Michigan University, Marquette, MI, United States; ^3^ Parks, Conservation, and Recreational Therapy Department, Slippery Rock University, Slippery Rock, PA, United States

**Keywords:** climate change, global warming, image database, environmental attitudes, emotion

## Abstract

Climate change is not only a scientific phenomenon, but also a cultural one. Individuals’ opinions on climate change are often based on emotion rather than on scientific evidence. Therefore, research into the emotional characteristics of the imagery that the non-expert public find relevant to climate change is important in order to build a database of effective climate change imagery, which can then be used by scientists, policymakers, and practitioners in mobilizing climate adaptation and resilience efforts. To this end, we collected ratings of relevance to climate change as well as emotional arousal and valence on 320 images to assess the relationship between relevance to climate change and the emotional qualities of the image. In addition, participants’ environmental beliefs were measured, to investigate the relationship between beliefs and image ratings. The results suggest that images rated highly relevant to climate change are higher in negative emotional valence and emotional arousal. Overall, images were rated as being more relevant to climate change by participants with higher pro-environmental disposition. Critically, we have compiled the mean relevance, valence, and arousal ratings of each of these 320 images into a database that is posted online and freely available (https://affectiveclimateimages.weebly.com; https://www.nmu.edu/affectiveclimateimages) for use in future research on climate change visuals.

## Introduction

In October 2018, the Intergovernmental Panel on Climate Change (IPCC) published a special report on the impact of a 1.5°C global temperature increase. The report, written by 91 authors, cites more than 6,000 scientific references and includes hundreds of scientific images illustrating the consequences of extreme weather, rising sea levels, diminishing Arctic ice, and irreversible ecosystem changes. The main argument in the IPCC’s special report is that limiting warming to 1.5°C will require that policymakers and practitioners make immediate and far-reaching changes in land, energy, industry, building, transportation, and urban policies in order to reduce human-caused emissions by 45% in the next 12 years. Arguably, over the past decade, climate change has turned from a scientific phenomenon to a cultural one (Nerlich et al., 2010) and individuals’ opinions are more likely based on values and emotions than on scientific evidence and data (Mckie and Galloway, 2007). Exploring the emotional characteristics of the imagery that the non-expert public find relevant to climate change is important in order to build a database of effective climate change imagery, which can then be used by scientists, policymakers, and practitioners in mobilizing climate adaptation and resilience efforts. Such a database is equally useful for experimental research and investigations of climate change imagery and messaging effectiveness.

Scholars in many disciplines have analyzed visual representations of the environment and specifically, images were used to communicate about climate change (e.g., Lester and Cottle, 2009; Hansen and Machin, 2013; Duan et al., 2017), but as Hansen and Machin (2013) noted, there is still a shortage in studies of the image’s impact on audiences and the audience’s affective responses to such images. Past studies have found that imagery involving melting glaciers, polar bears, and destruction from natural disasters are most commonly reported by participants when asked to mentally visualize climate change (Nicholson-Cole, 2005; Leiserowitz, 2006; O’Neill and Nicholson-Cole, 2009), and that images depicting dramatic outcomes of climate change, such as dried up lakes or flooding, are most commonly reported as making climate change seem most important (O’Neill and Nicholson-Cole, 2009; O’Neill et al., 2013). The emotional qualities of these images were only directly measured in one of these studies, which analyzed emotional valence, meaning how “positive” or “negative” a stimulus is perceived (Posner et al., 2005). Participants rated the valence of their mental representation of climate change from −5 to +5, and the average valence rating was reported for each of the most common category of climate images (Leiserowitz, 2006). However, participants only rated the valence of their own mental imagery—every “image” is not rated by every participant, limiting the objectivity of these images and their valence scores.

Emotion is not directly, objectively measured in the majority of past research on climate change imagery. Images’ affect as described in the research is based on participants’ reactions to and descriptions of the images. For example, images of climate change impact is determined to be negative by participants describing them in interviews as “very scary” (O’Neill and Nicholson-Cole, 2009) and “horrific” (O’Neill et al., 2013). Ranking images based on these descriptions becomes impossible due to the subjectivity: it is unclear whether a “horrific” image has greater negative affect than a “very scary” image, while an image rated as −5 in affect is certainly more negative than an image rated as −1 (e.g., Leiserowitz, 2006).

There is also no way to replicate the climate change imagery evaluated in these studies, as often the imagery was mental visualizations by participants (e.g., Leiserowitz, 2006; O’Neill and Nicholson-Cole, 2009). When the images were collected from permanent sources for participants to assess (e.g., O’Neill and Hulme, 2009; O’Neill et al., 2013), they were not made accessible for readers to view let alone use. Research on imagery in newspaper stories about climate change has also not made the images in question accessible (e.g., DiFrancesco and Young, 2011; O’Neill, 2013; Hart and Feldman, 2016; Duan et al., 2017). This lack of standardization of images and their limited accessibility for use in experiments thus calls for a dedicated stimulus set for climate images, similar to the Face Place (as used in Righi et al., 2012) and International Affective Picture System (IAPS; Lang et al., 1997).

A library of images called Climate Visuals was recently published containing over 1,000 photographs best suited for use in climate change communications based on non-expert perceptions (Chapman et al., 2016). Participants were interviewed on their opinions of 49 climate-related images, chosen with help of experts, in international focus groups (Chapman et al., 2016). A large sample of international participants were surveyed on 30 of those images, rating them on a number of variables, including affect on a scale of −5 to +5 (Chapman et al., 2016). The results from this study showed that participants found typical climate imagery—melting ice, polar bears—to be “cliché,” though they were the most understood and most easily recognizable as being climate images in the survey portion (Chapman et al., 2016). Images depicting climate change solutions were generally rated as having positive affect but decreased personal motivation to make climate-beneficial behavioral changes, while images of climate change causes were generally rated as having negative affect but more likely to be shared by and motivate participants to make personal changes (Chapman et al., 2016). Chapman and colleagues therefore recommend images depicting non-staged people and large-scale causes of climate change over commonly used visual themes like melting ice when adding visuals to climate change communications (Corner et al., 2015). The images that make up the Climate Visuals public database thus reflect these qualities determined through their research to best communicate climate messages.

The Climate Visuals library is intended for use by climate change advocacy organizations, bloggers, and journalists, (Chapman et al., 2016) and was designed accordingly. For experimental research, however, it is less ideal: only 30 images used in the survey phase of the experiment were rated for affect (i.e., valence), and participants only rated six of those images each (Chapman et al., 2016). Although images were rated on emotional valence, the arousal dimension (i.e., excitement or physiological arousal) was not assessed. Therefore, potential differences in image processing related to valence vs. arousal cannot be determined with this existing database. In addition, all images were high relevance images. No low relevance images underwent the same rating procedure. The images used in the survey—or a similar image, when usage rights were not obtained—along with their mean ratings, including affect, are presented in a downloadable appendix on the Climate Visuals website^1^. As in past studies, not every image was rated by every participant, and a majority of the images included in the library were not rated at all.

A different database is necessary for further experimental research on climate imagery in order to promote consistency in stimuli used, so that the direct comparison of results using these stimuli is possible. We have created such a database. We began by collecting a large number of images (see Method for details) and then invited non-expert participants to rate the images’ affective qualities and their relevance to climate change. Measuring images’ climate relevance is necessary to have both experimental and control stimuli in this database for use in future research. Images’ affect was measured using a dimensional model of emotion, with arousal (calming/exciting) and valence (negative/positive) as the two dimensions on a scale from 1 to 9, as this has been determined to be most efficient and more objective than using common emotion words like “happy” or “sad” (Russell, 1980). This model is supported by other similar image-rating tasks such as the IAPS (Lang et al., 1997).

Additionally, we measured participants’ environmental beliefs. Past studies have tended not to record participants’ environmental beliefs or opinions (e.g., O’Neill and Nicholson-Cole, 2009) or not report on them in relation to their findings on climate imagery (e.g., Leiserowitz, 2006; O’Neill and Hulme, 2009; O’Neill et al., 2013). Only Chapman and colleagues surveyed participants’ skepticism toward climate change and reported finding that skepticism impacted participants’ feelings toward images of climate solutions (Chapman et al., 2016). Individual differences in environmental beliefs and opinions (whether broadly or specifically toward climate change) are important to account for, as individuals’ environmental concern has been shown to be a positive predictor of self-reported pro-environmental behavior (e.g., Steel, 1996; Olli et al., 2001; Clark et al., 2003; Kim and Choi, 2005) and have a positive relationship with environmental policy support and adoption (Brace et al., 2002; Johnson et al., 2005; Dietz et al., 2007). Participants’ environmental beliefs were measured using the New Ecological Paradigm Scale (NEP; Dunlap et al., 2000), as it is recommended as the standard for this measure (Hawcroft and Milfont, 2010) and has been widely used both in the US and internationally (see Dunlap, 2008).

### Hypotheses

This study aims to create an accessible database of images for use in climate research, with image ratings performed by a sample of non-experts in order to be more suited for use with non-expert audiences. Images are rated on three variables—relevance to climate change, arousal, and valence—and we predict:

1. images rated highly relevant to climate change will also be more likely to be rated as being high-arousal and low-valence, given how images depicting dramatic, negative themes have consistently been found to be more salient to people regarding climate change (Leiserowitz, 2006; O’Neill and Nicholson-Cole, 2009; O’Neill et al., 2013) and
2. there will be a positive relationship between beliefs about the environment and image ratings of relevance, arousal, and valence, given the NEP Scale’s predictive validity in correlating positively with respondents’ other environmental views (Dunlap et al., 2000).

## Materials and Methods

### Participants

Participants for this experiment consisted of 67 males (*n* = 30) and females (*n* = 37) between the ages of 18 and 38 years old (*M* = 20.373, *SD* = 3.789), with normal or corrected-to-normal vision. They were recruited primarily through undergraduate psychology classes on Northern Michigan University’s campus, receiving course credit for their participation. Informed consent was obtained from participants before beginning the experiment, and the research protocol was approved by the Institutional Review Board of Northern Michigan University.

### Procedure

#### Image-Rating Stimuli

A total of 320 images were gathered from a Google search using the following terms involving climate change: (1) “climate change,” (2) “climate change causes,” (3) “climate change solutions,” (4) “climate change negative,” and (5) “climate change positive.” Search results were filtered to only include high resolution images not containing clipart that were labeled for reuse. We selected the top 100 images from each of these five searches, which resulted in 500 images. Images that were redundant across multiple searches were only included once and images of artwork were removed. The resulting number of images included was 320.

#### Image-Rating Task

**Figure 1:**
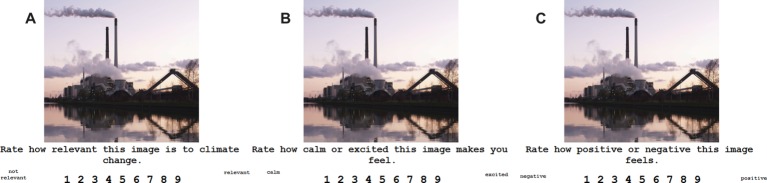
Image-rating task screens. Participants were asked to rate the stimulus on its **(A)** relevance to climate change, **(B)** arousal, and **(C)** valence. Participants rated each stimulus on each variable before the next stimulus was presented.

The image-rating task was designed using E-Prime2 software (Psychology Software Tools, Pittsburg, PA). The image-rating task began with participants seated 59 cm from the computer screen. They were told that they were going to be shown pictures and asked to rate them on a scale of 1–9 for each of the variables given on the screen. Images were displayed above a question and rating scale for each variable. For each image, participants were asked how relevant or irrelevant it was to climate change (relevance), how calming or exciting it made participants feel (arousal), and how negative or positive the image appeared (valence), in that order consistently. Each image was presented for each variable scale before the next image was shown in a random order (Figure 1). Participants used the computer’s keyboard number pad to input their ratings. The task was not timed, and took participants approximately an hour to complete.

#### Questionnaire

Immediately after finishing the image rating task, participants were asked to complete the New Ecological Paradigm Scale (Dunlap et al., 2000). This was done after the image-rating task in order to avoid potentially priming participants to think about environmental issues before rating the images on climate change relevance. Their responses were given on a 5-point Likert scale from “strongly agree” to “strongly disagree.” The items on this questionnaire are meant to gauge the participants’ views on the environment as a whole, and human beings’ impact on the Earth (for full scale, see Dunlap et al., 2000). The 15-item questionnaire with 5-point response scale is used for this study as recommended based on meta-analysis of 30 years of NEP Scale usage (Hawcroft and Milfont, 2010). This questionnaire has high internal consistency, *α* = 0.83, as determined through a representative sample of Washington state residents (Dunlap et al., 2000).

### Data Analysis

#### Image-Rating Task

The mean rating of each variable (relevance, arousal, and valence) was collected for each of the 320 images. Pearson correlations were performed to test for positive relationships between (1) relevance and arousal, (2) relevance and valence, and (3) arousal and valence. The significance level was set to *p* < 0.05, two-tailed.

#### Questionnaire

Participants’ responses from 1 to 5 on the NEP Scale were made into composite scores, with reverse scoring performed for even numbered items as detailed by Dunlap et al. (2000). The highest possible composite score is 75, indicating greater pro-environmental beliefs as well as interest in and concern for the environment, particularly its ability to be disrupted by human beings (Dunlap et al., 2000). The lowest possible composite score is 15, indicating feelings of human beings’ dominance over nature and less concern for the environment, or lesser pro-environmental beliefs (Dunlap et al., 2000).

#### Image-Rating Task + Questionnaire

The relationship between participants’ image ratings and NEP Scale questionnaire responses is also of interest. In order to accurately determine the potential relationship between participants’ ratings of climate image relevance and their beliefs about the environment, each participant’s average ratings for relevance, arousal, and valence of the 10% of images determined to be *most* relevant and the 10% of images determined to be *least* relevant (*n*_total_ = 64) were correlated with their response for each NEP Scale item using Pearson correlations. The significance level was set to *p* < 0.05, two-tailed. Correlation strength is interpreted based on Cohen’s conventions (i.e., small *r* = |0.1|, medium *r* = |0.3|, and large *r* = |0.5|; Cohen, 1988).

## Results

### Image-Rating Task

**Figure 2:**
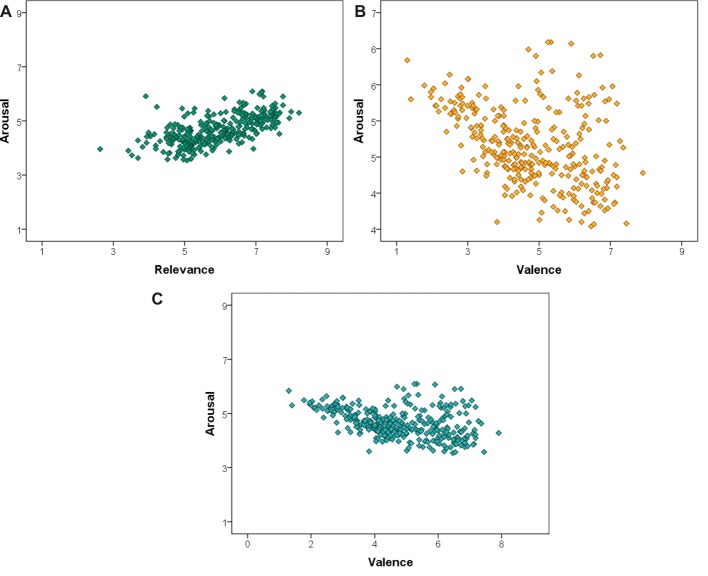
**(A)** There was a strong, positive correlation between images rated high in relevance to climate change and images rated high in arousal. **(B)** There was a moderately strong, negative correlation between images that were rated high in relevance to climate change and images that were rated low in valence. **(C)** There was a moderate, negative correlation between images that were rated low in valence and images that were rated high in arousal.

The average relevance ratings of the images (*M* = 5.909, *SD* = 1.033) were positively correlated with the average arousal ratings of the images (*M* = 4.651, *SD* = 0.531), *r*(318) = 0.621, *p* < 0.001. The correlation was strong, showing that the images determined to be most relevant to climate change were also rated by participants as being highly arousing, or exciting to look at (Figure 2A).

The average relevance ratings were also negatively correlated with the average valence ratings of the images (*M* = 4.793, *SD* = 1.382), *r*(318) = −0.432, *p* < 0.001. The correlation is moderate, and shows that the images most relevant to climate change were also rated as having low valence, or as being very negative (Figure 2B).

There was also a moderate, negative correlation between the average arousal ratings and the average valence ratings of the images, *r*(318) = −0.394, *p* < 0.001, showing that the images that were rated as most exciting were also some of the most negative (Figure 2C). This is not surprising given the results of similar image ratings in the IAPS, which initially reported having very few images, which were rated as unpleasant yet also un-arousing (Lang et al., 1997).

**Figure 3:**
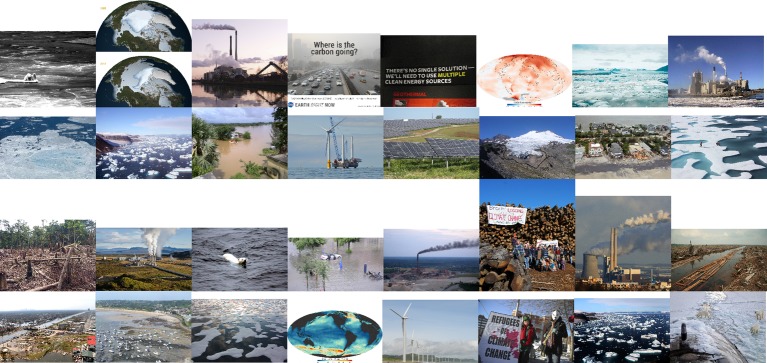
The 32 images rated highest in relevance to climate change, read from top left to bottom right.

**Figure 4:**
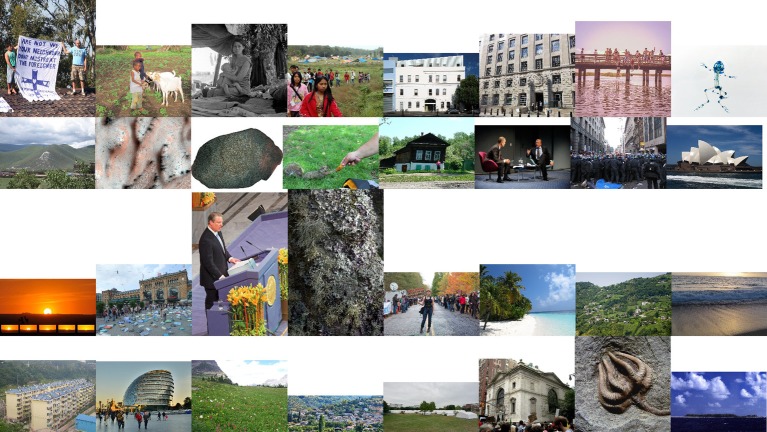
The 32 images rated lowest in relevance to climate change, read from bottom right to top left.

We also selected the 10% *most relevant* images (*n* = 32; *M* = 7.537, *SD* = 1.304) and the 10% *least relevant* images (*n* = 32; *M* = 4.121, *SD* = 1.678) to be used in further correlation analyses. Common themes depicted in the *most relevant* images were polar bears, ice floes, industrial smog, and outcomes of natural disasters (Figure 3), while common themes depicted in the *least relevant* images were landscapes, buildings, and people (Figure 4).

### Questionnaire

Of the 67 total participants, 62 completed the NEP Scale questionnaire following the image rating task. The highest score recorded in this experiment was 74 and the lowest was 32, out of a maximum 75 and minimum 15 (*M* = 53.177, *SD* = 8.434). Internal consistency for this sample was high, *α* = 0.84.

### Image Ratings + Questionnaire Responses

**Figure 5:**
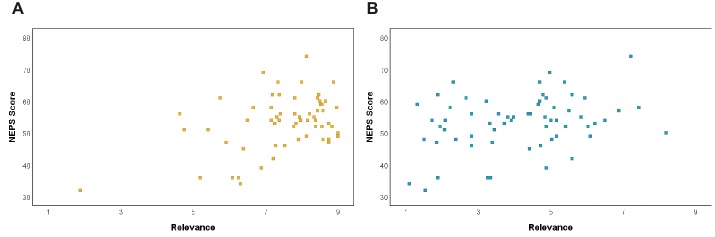
**(A)** There was a strong, positive relationship between the average relevance ratings of the images determined to be *most relevant* to climate change and participants’ score on the NEP Scale. **(B)** There was a moderate, positive relationship between the average relevance ratings of the images determined to be *least relevant* to climate change and participants’ score on the NEP Scale.

There were two significant relationships found between image ratings and questionnaire responses. There was a moderate positive correlation between the average relevance ratings of the *most relevant* images and participants’ NEP Scale scores, *r*(60) = 0.419, *p* = 0.001. This shows that participants with more pro-environmental beliefs or more interest in the environment were more likely to give the *most relevant* images their high relevance ratings (Figure 5A). There was also a moderate positive correlation between the average relevance ratings of the *least relevant* images and participants’ NEP Scale scores, *r*(60) = 0.31, *p* = 0.003. This shows that participants with more pro-environmental beliefs, again, more likely to give the *least relevant* images their low relevance ratings (Figure 5B).

There were no significant correlations between environmental beliefs and arousal scores, *r*(60) = 0.001, *p* = 0.994, or valence scores, *r*(60) = −0.174, *p* = 0.175, for the *most* relevant images. Nor were there significant correlations between environmental beliefs and arousal scores, *r*(60) = −0.041, *p* = 0.751, or valence scores, *r*(60) = 0.005, *p* = 0.969, of the *least* relevant images. This suggests that participants’ ecological views may not have been related to their opinions on the emotional qualities of the images^2^, only their opinions on the images’ relevance to climate change.

## Discussion

From the image-rating task, relevance to climate change and arousal were significantly positively correlated, as were relevance and valence. Arousal and valence were significantly negatively correlated. Therefore, images that were rated as highly relevant to climate change also tended to be rated as highly arousing and low in valence, while images that were rated high in arousal tended to be rated as low in valence in general. The 64 images that were rated *most* and *least* relevant to climate change were then selected for further correlation analyses.

We found from the questionnaires that participants had varying levels of pro-environmental beliefs according to their scores on the NEP Scale, with high scores indicating greater pro-environmental beliefs and interest in the environment and low scores indicating less interest and fewer pro-environmental beliefs. Pro-environmental beliefs were found to be significantly positively correlated with the relevance scores of the 32 *most* relevant images and the 32 *least* relevant images, but not with arousal scores or valence scores. Higher pro-environmental beliefs were associated with higher relevance scores, among both the images rated *most* relevant to climate change and those rated *least* relevant to climate change.

### Hypothesis 1

Our first hypothesis was supported by our findings. The strong positive relationships between relevance and arousal show that generally, the images determined to be most relevant to climate change were also determined to be the most arousing or exciting. The negative relationship between relevance and valence showed that the images most relevant to climate change also tended to be the most negative images, emotionally. Exciting, emotionally negative images being rated as the most relevant to climate change in our study is also in line with previous research which has indicated that non-expert individuals tend to find alarming and upsetting imagery most salient when thinking about climate change and global warming (Leiserowitz, 2006; O’Neill and Nicholson-Cole, 2009; O’Neill et al., 2013).

The primary subject matter of the images found to be *most* relevant to climate change in this study included ice, outcomes of natural disasters, and industrial buildings or smog (see Figure 3). According to previous research, these three themes were among those that first came to mind to individuals thinking about climate change and global warming (Leiserowitz, 2006; O’Neill and Nicholson-Cole, 2009), and were also determined by individuals to make climate change seem most important (O’Neill and Nicholson-Cole, 2009; O’Neill et al., 2013). Images depicting the outcomes of natural disasters exemplify what is meant by high-arousal/low-valence imagery in this study: they are dramatic and distinctly negative in affect.

The image rated most relevant to climate change overall depicted polar bears on small ice floes in the water, though only three of the 32 *most* relevant images depicted polar bears. Polar bears are a popular visual symbol for climate change (Manzo, 2010), and in one study they were the climate icon participants were most drawn to and able to understand (O’Neill and Hulme, 2009). While this type of imagery was found to be “cliché” or overused by participants surveyed by Chapman et al. (2016), it was also determined to be easy to understand as relating to climate change. This is most important to our database—images included are intended to be used in experimental research on climate change, thus images need to be easily recognizable as climate-relevant to participants. We have also chosen not to investigate participants’ feelings of self-efficacy regarding the images as some past research has done, as those results indicate that imagery rated high in self-efficacy, or the ability to enact change against climate change, tend to be rated low in climate-relevance (e.g., O’Neill and Nicholson-Cole, 2009; O’Neill et al., 2013). Images’ relevance to climate change was prioritized in this study, though others using this database in continued research are encouraged to investigate measures of self-efficacy.

The subject matter of the images found to be *least* relevant to climate change in this study included landscapes or nature, people, and buildings (see Figure 4). Past research has found that images with similar themes, such as buildings and landscapes, have been ranked as making climate change seem least important (O’Neill and Nicholson-Cole, 2009), as have images of people, particularly if they are identifiable (O’Neill et al., 2013). This may explain, then, why a photo of US politician Al Gore was determined by our participants to be one of the least relevant images to climate change, despite having won the 2007 Nobel Peace Prize jointly with the IPCC for his work spreading awareness about the anthropogenic nature of climate change (Gibbs and Lyall, 2007). The ages of our participants (*M* = 20.373, *SD* = 3.789) may also be salient, however, as it is possible that they were simply unfamiliar with Gore.

The subject matter present in the images that were rated *most* and *least* relevant to climate change in this study shows that the high-arousal/low-valence, dramatic and negative, imagery that was correctly predicted to be most relevant to climate change was mainly represented by scenes of natural disaster outcomes. Images of ice floes and industrial buildings with smog were also common themes in this category, although an image of polar bears on ice was rated as the image most relevant to climate change overall. The images rated *most* relevant consisted primarily of causes and consequences of climate change, with potential solutions represented very little, which replicates previous findings (e.g., Leiserowitz, 2006; O’Neill and Nicholson-Cole, 2009; O’Neill et al., 2013) and suggests that the high-arousal/low-valence combination of emotional characteristics is particularly necessary for images to beseen as relevant to climate change. (However, potential solutions were represented much more in the top 50% of climate-relevant images).

Meanwhile, many of the images in the *least* relevant category seem self-explanatory, such as a photo of a squirrel. However, images of people are frequently found not to be salient to climate change, as represented in our findings, despite being the most common category of picture attached to news media coverage of climate change (both in print and digitally; DiFrancesco and Young, 2011; O’Neill, 2013) and endorsed as visuals for climate communication (Corner et al., 2015). While this may appear to contradict past results like those of the Climate Visuals library (Chapman et al., 2016), depictions of people in the top 50% of climate-relevant images are generally in line with the recommendations used to compile the Climate Visuals library: these images are mostly of groups of protesters, photos which show “real people” in situations that are not “staged” (Corner et al., 2015). So, while images depicting people were more likely to be rated as irrelevant to climate change in general, those that were rated more relevant to climate change possessed the attributes that current research recommends when portraying climate change.

### Hypothesis 2

Our second hypothesis was partially supported by our findings. There were significant positive correlations between the participants’ environmental beliefs (as determined by NEP score) and their relevance ratings toward both the *most* and *least* relevant images, so it appears that environmental views were linked to participants’ ratings of the images’ relevance to climate change. Individuals with greater pro-environmental beliefs appeared more likely to give high relevance ratings to the images that were subsequently determined to be *most* relevant to climate change, as well as appearing to be more likely to give higher relevance ratings to the images that were subsequently determined to be *least* relevant to climate change. This seems to show that individuals who were highly concerned about the environment tended to find the images overall more relevant to climate change than participants who were not as concerned about the environment.

On the other hand, as there were no significant relationships between environmental beliefs and arousal or valence scores of the images both *most* and *least* relevant to climate change, it appears that we were not correct in predicting that individuals’ environmental views are related to their ratings of images’ affective characteristics. Meaning, exciting and negative images seemed exciting and negative to participants regardless of how they felt about the environment.

These findings address the lack of consideration for environmental beliefs in previous research done on climate change imagery. For example, some previous studies have recorded participants’ attitudes toward climate change, but have not investigated these attitudes and how participants rated images as salient or self-efficacious regarding climate change (O’Neill and Hulme, 2009; O’Neill et al., 2013). In both cases, a sample with varied climate attitudes was desired and achieved, yet appears not to have been applied to the image ratings. Our findings that individuals’ environmental beliefs, as measured by the NEP scale, were related to their ratings of the images as being relevant to climate change partially supports our prediction for this study, and also attempts to fill a gap in literature on climate imagery.

### Strengths and Limitations

Of course, there were limitations to our study that may have attributed to some of our findings, or lack thereof. Given that scores on the New Ecological Paradigm Scale have been positively correlated with both age and education (Dunlap et al., 2000; Hawcroft and Milfont, 2010), if only slightly, our student sample may have had an effect on the image rating results. However, student samples have been found to be quite comparable to more representative samples when using the NEP (Hawcroft and Milfont, 2010). In addition, although we report the relationship between environmental disposition and image ratings, future research could specifically assess the relationship between climate change attitudes and ratings for climate images. There are also some limitations regarding the images that we used for this study. Specifically, results from the image-rating task have shown that very few images have been rated as both low-arousal and low-valence. As these images and their ratings are being used in a database, we would prefer to have an equal spread of images with all combinations of affective characteristics so that future users can choose images with qualities that meet their needs. However, this was also a problem encountered by IAPS in its early stages (Lang et al., 1997). In addition, open access images of climate change may be of lesser quality than those of professional photographers that restricted usage rights (although it should be noted that our search was filtered to only include high resolution images). These limitations could potentially be rectified by expanding our sample size, both of participants and of images. Future replication of this experiment using these same images with a wider, more diverse range of participants is necessary.

There were also many strengths to this study, most notably our appeal to climate change non-experts, both as participants and as an audience for climate change communication. Scholars recommend that this communication be shaped according to the audience (Leiserowitz, 2006), appealing to what they find meaningful (Nerlich et al., 2010). Given that climate change communication and scientific communication in general, often uses visual imagery to illustrate these messages (Trumbo, 1999; Nicholson-Cole, 2005), we chose to explore what visual imagery is meaningful to our non-expert participants. It is also recommended that climate communication should involve non-experts’ understanding, emotions, and behavior (Ockwell et al., 2009), which we have done by having participants rate the images’ relevance to climate change (understanding) and arousal and valence (emotions), and then surveying their environmental beliefs (behavior). In this way our images and their ratings should be particularly suited for future use with other non-expert audiences.

Our study also adds further evidence to support an apparent gap between what imagery non-expert individuals feel is relevant to and best represents the importance of climate change, and what imagery is used by news media when covering climate change. For example, print and digital newspapers in the Canada, US, the UK, and Australia all primarily use images of people, particularly politicians, to accompany these articles (DiFrancesco and Young, 2011; O’Neill, 2013), despite our study finding these types of images most commonly rated least relevant to climate change, and another finding them to make climate change seem the least important across participants in three countries (O’Neill et al., 2013). As this type of media seems to be most non-experts’ primary source of information on climate change (Wilson, 2000; Nicholson-Cole, 2005; Sundblad et al., 2009), this gap is concerning. Clearly news media’s climate communications are not attending to what their audiences find important in ways recommended by scholars in the field, and our results seem to add support to this.

As scientific language and colloquial language often use the same words with different meanings, the language used in climate change communication can create confusion rather than convey the facts, depending on the audience (Nerlich et al., 2010). Because of this, we utilized commonly-used language in our rating systems (calming or exciting, negative or positive) that still properly expressed our objective variables (arousal, valence), lessening the opportunities for misinterpretation by non-expert participants.

The objectiveness of these variables is another strength of our study; by using quantitative ratings of relevance to climate change, arousal, and valence, these images can be definitively measured against each other according to each variable. Past studies have only ranked images based on relevance, though not with numerical value (O’Neill and Hulme, 2009; O’Neill and Nicholson-Cole, 2009; O’Neill et al., 2013), with only subjective descriptions of the images’ emotional qualities. Additionally, these ratings were performed on each image by each participant, as opposed to each participant rating only their own personally-relevant climate imagery on its affect (Leiserowitz, 2006). Because of this, our images’ ratings come from a larger sample, and are thus more objective.

Since this is one of the first research-generated databases of visual experimental stimuli related to climate change, previous research has relied on mental imagery elicited from participants (Leiserowitz, 2006; O’Neill and Nicholson-Cole, 2009) or images gathered from expert scientific sources (O’Neill and Hulme, 2009) and newspapers (O’Neill et al., 2013), none of which have been made available online or are included in publications. A recent climate image database has since emerged, but is not appropriate for experimental research both in subject matter and in image data availability: images in the Climate Visuals library have been selected based on their ability to promote audience engagement and as such the library primarily contains photographs of people. While these are useful for climate communications and media, we have found that this type of image alone is not seen as relevant to climate change, and thus are not appropriate for use as stimuli in climate change research where they would be devoid of any context. In addition, many Climate Visuals photos are available online with descriptions of the photos’ contents, but do not include any data from the creators’ research, such as how the image made participants feel about climate change, as not every image was rated in the initial study. While there are links for acquiring each image included as well, not all images are available for free or to be reused, limiting the library’s utility as a stimulus set.

Because of this, our database is accessible online with all images available for download and reuse, and with climate-relevance and affective characteristic ratings shown for each image^3^. These ratings have been completed by a non-expert audience in order to best be used as experimental (and control) stimuli in further research on climate change imagery, with climate-relevance determined based on the image alone. Our rating system ensures that any future studies using these images will have the ability for direct comparison, and eliminates any confounds in comparing the results of two studies due to different stimulus sets used. Understanding the cognitive processes associated with climate change (including those related to processing climate images) is important for understanding people’s climate change relevant behavior. For example, we recently used stimuli from this database to demonstrate that images with high relevance to climate change facilitate reaction times and capture observers’ attention compared to low relevance images and this attentional bias is heightened in individuals with pro-environmental attitudes (Carlson et al., 2019).

## Conclusion

Our goal for this study was to gather objective, quantitative data on how individuals’ viewed the affective characteristics of climate-related imagery in order to create an accessible database of stimuli for use in experimental research on climate imagery. We achieved this goal while also supporting the findings of previous, similar studies on this subject that there are common subjects and emotional aspects that are most salient to people when visualizing climate change. We also found that individuals’ interest in the environment has effects on the way they rate images as being relevant or irrelevant to climate change. Non-expert opinions were prioritized in this study, and it was carried out in such a way that it should generalize to non-expert audiences viewing and using these images in future studies.

## Ethics Statement

This study was carried out in accordance with the recommendations of Northern Michigan University Institutional Review Board with written informed consent from all subjects. All subjects gave written informed consent in accordance with the Declaration of Helsinki. The protocol was approved by the Northern Michigan University Institutional Review Board.

## Author Contributions

JC, JT, and SD conceived the idea for the study and designed the study. BL collected and analyzed the data. BL drafted the manuscript. All authors critically revised the initial draft and approved the final version.

### Conflict of Interest Statement

The authors declare that the research was conducted in the absence of any commercial or financial relationships that could be construed as a potential conflict of interest.

## Footnotes

^1^www.climatevisuals.org

^2^Analyses of curvilinearity (i.e., a quadratic trend) between environmental beliefs and valence scores of the *most* relevant images (*p* = 0.87) and the *least* relevant images (*p* = 0.14) were also not significant. This indicates no relationship between environmental beliefs and extreme emotionality, either positive or negative.

^3^https://affectiveclimateimages.weebly.com; www.nmu.edu/affectiveclimateimages
